# Polyenylphosphatidylcholine alleviates cardiorenal fibrosis, injury and dysfunction in spontaneously hypertensive rats by regulating Plpp3 signaling

**DOI:** 10.3389/fcvm.2024.1458173

**Published:** 2024-09-30

**Authors:** Yongqiao Zhang, Jiayi Ma, Feng Wei, Jiaxin Gong, Li Zhuang, Ningzhi Zhang, Zhaoqiang Cui

**Affiliations:** ^1^ Department of Cardiology, Zhongshan Hospital, Fudan University, Shanghai Institute of Cardiovascular Diseases, Shanghai, China; ^2^ The Fifth Clinical College of Medical, Xinxiang Medical University, Henan, China; ^3^ Department of Cardiology, Shanghai Changning Tianshan Traditional Chinese Medicine Hospital, Shanghai, China

**Keywords:** hypertension, Plpp3, polyenylphosphatidylcholine, spontaneously hypertensive rats, NF-*κ*B

## Abstract

**Objective:**

Polyenylphosphatidylcholine (PPC), a significant therapeutic agent for liver repair, exhibits potent antioxidant and anti-inflammatory properties. Nonetheless, its impact on hypertension and hypertensive vascular diseases requires clarification. Our objective was to elucidate the protective role and mechanism of PPC in a spontaneously hypertensive rat model.

**Materials and methods:**

Male WKY and SHRs were randomly assigned to four groups: WKY control, SHRs control, SHRs treated with Telmisartan (SHR-TS), and SHRs treated with PPC (SHR-PPC). Blood pressure was monitored biweekly during the treatment. Histological analyses assessed aortic vascular remodeling and cardiac and renal injuries. RNA-seq was performed on vascular smooth muscle cells (VSMCs) isolated from WKY or SHRs, and protein levels of target genes were quantified using Western blotting.

**Results:**

In a dose-dependent screening test, we confirmed that PPC (200 mg/kg**/**day) effectively reduced blood pressure in SHRs. Treatment with PPC also mitigated cardiac and renal injury in SHRs by attenuating hypertrophy and fibrosis. Compared to WKY rats, SHRs exhibited increased intima thickness, reduced vascular tone, and heightened aortic fibrosis; however, PPC treatment significantly reversed vascular remodeling. Analysis of RNA-seq data revealed that downregulated genes were enriched in inflammation and oxidative stress pathways based on GO and KEGG enrichment analyses. PPC markedly inhibited genes such as Rela, Relb, Nfkb2, and others involved in the NF-*κ*B pathway. Given PPC's influence on glycerophospholipid synthesis and metabolism, and its role in NF-*κ*B-mediated transcription affecting oxidative stress and inflammation, changes in the PLAs, PLPs, and PLPPs families were analyzed in PPC-treated VSMCs. Among these, PPC notably inhibited Plpp3. Importantly, overexpression of Plpp3 significantly reversed the protective effects of PPC on hypertension-related cardiac and renal injuries, vascular fibrosis, remodeling, and tension.

**Conclusion:**

We identified a new protective role for PPC in mitigating cardiac and renal injuries associated with hypertension, as well as in preventing aortic fibrosis and remodeling. Targeting the NF-*κ*B/Plpp3 pathway may offer a promising therapeutic strategy for treating vascular diseases related to hypertension.

## 1 Introduction

Lipid and fatty acid metabolism significantly influence vascular integrity and function (1). Impaired lipid and fatty acid metabolism compromise cell membrane phospholipids, increasing oxidative and inflammatory stress, closely correlating with conditions such as aortic sclerosis, remodeling, and pulmonary artery hypertension (2–4). The impact of lipid metabolism on vascular endothelial cells has been extensively researched, linking lipid accumulation and impaired fatty acid oxidation to hypertension-related conditions (5). Situated in the subintimal layer of blood vessels, vascular smooth muscle cells (VSMCs) maintain vascular structural integrity and control aortic contraction and relaxation. However, dysregulated VSMC proliferation and migration contribute to vascular injury, aortic stiffness, and pathological hypertension (6–8). Swiatlowska P. first reported that hypertensive pressure alone induces phenotypic switching of VSMCs through disrupted lipid metabolism and rapid lipid droplet accumulation, resulting in VSMC-foam cell formation (9).

Phosphatidylcholine is a ubiquitous cellular molecule and a key source of choline in the human body. Polyenylphosphatidylcholine (PPC) is a classic hepatoprotective drug, widely used in the treatment of liver metabolic disorders and inflammatory hepatitis (10). PPC has a strong effect on anti-inflammation and anti-oxidative stress (11). Recently, a study by the Markin S. group reported that PPC significantly promotes vascular cholesterol efflux and effectively delays the progression of atherosclerosis (12). However, the role of PPC in hypertension and hypertension-related vascular pathology, particularly its impact on VSMCs metabolism and function, remains unclear.

In this study, we examined the effects of PPC on SHRs *in vivo* and on cultured VSMCs derived from SHRs *in vitro*. The results showed that PPC treatment effectively reduced blood pressure and protected organs. Moreover, PPC demonstrated superior effectiveness over Telmisartan (TS) in preventing inflammation and oxidative stress in VSMCs. Mechanistically, we discovered that PPC decreased reactive oxygen species (ROS) production, suppressed NF-*κ*B activity, and inhibited the Plpp3-mediated inflammatory pathway in VSMCs. This led to reduced secretion of inflammatory cytokines such as TNF-α and IL-1β, subsequently preventing VSMC proliferation and migration, as well as organ fibrosis. Our study highlights lipid metabolic dysfunction in hypertension-related diseases, suggesting Plpp3 as a potential therapeutic target.

## 2 Materials and methods

The detailed materials and methods can be found in the online Supplementary Material.

## 3 Results

### 3.1 PPC reduced the elevated blood pressure in SHRs

**Figure 1:**
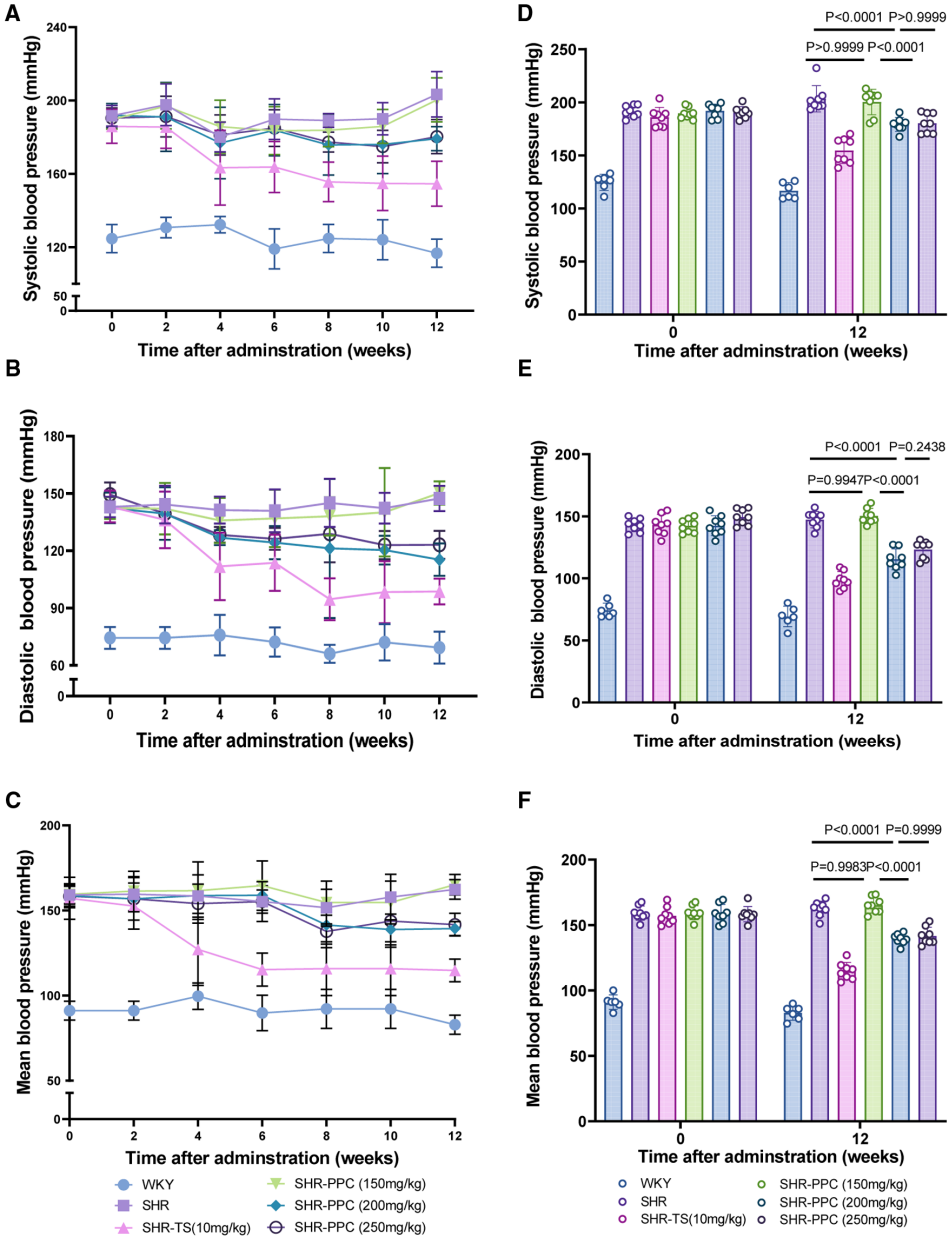
PPC mitigated the elevation of tail arterial pressure in SHR rats. **(A–C)** SBP, DBP or MBP was determined in WKY or SHR rats during 12 weeks with the treatment of PPC or TS. **(D–F)** Statistical analysis of SBP, DBP or MBP changes at the beginning (0 W) and end (12 W) of the experiment in WKY and SHR rats with or without treatment of PPC or TS. Data were presented as mean ± SD (*n* = 6–8 per group), SBP, systolic blood pressure; DBP, diastolic blood pressure; MBP, mean blood pressure.

To investigate the impact of PPC on blood pressure regulation, 2-month-old SHRs were administered three different doses of PPC over a 12-week period. Compared to WKY rats, SHRs exhibited significantly elevated SBP, DBP, or MBP (Figure 1). Interestingly, compared to TS, PPC treatment also resulted in significant reductions in blood pressure. At a dosage of 150 mg/kg/day, reductions were observed at 1.51, −1.97, and −1.74%, respectively, compared to untreated SHRs. At 200 mg/kg/day, reductions were more pronounced, with decreases of 11.89, 21.71, and 14.14%, respectively. At 250 mg/kg/day, reductions were 11.34% for SBP, 16.43% for DBP, and 12.73% for MAP compared to the SHRs control group. The identified optimal dosage was 200 mg/kg/day, which will be employed in subsequent experiments.

### 3.2 PPC attenuated chronic hypertensive heart and kidney injury in SHRs

**Figure 2:**
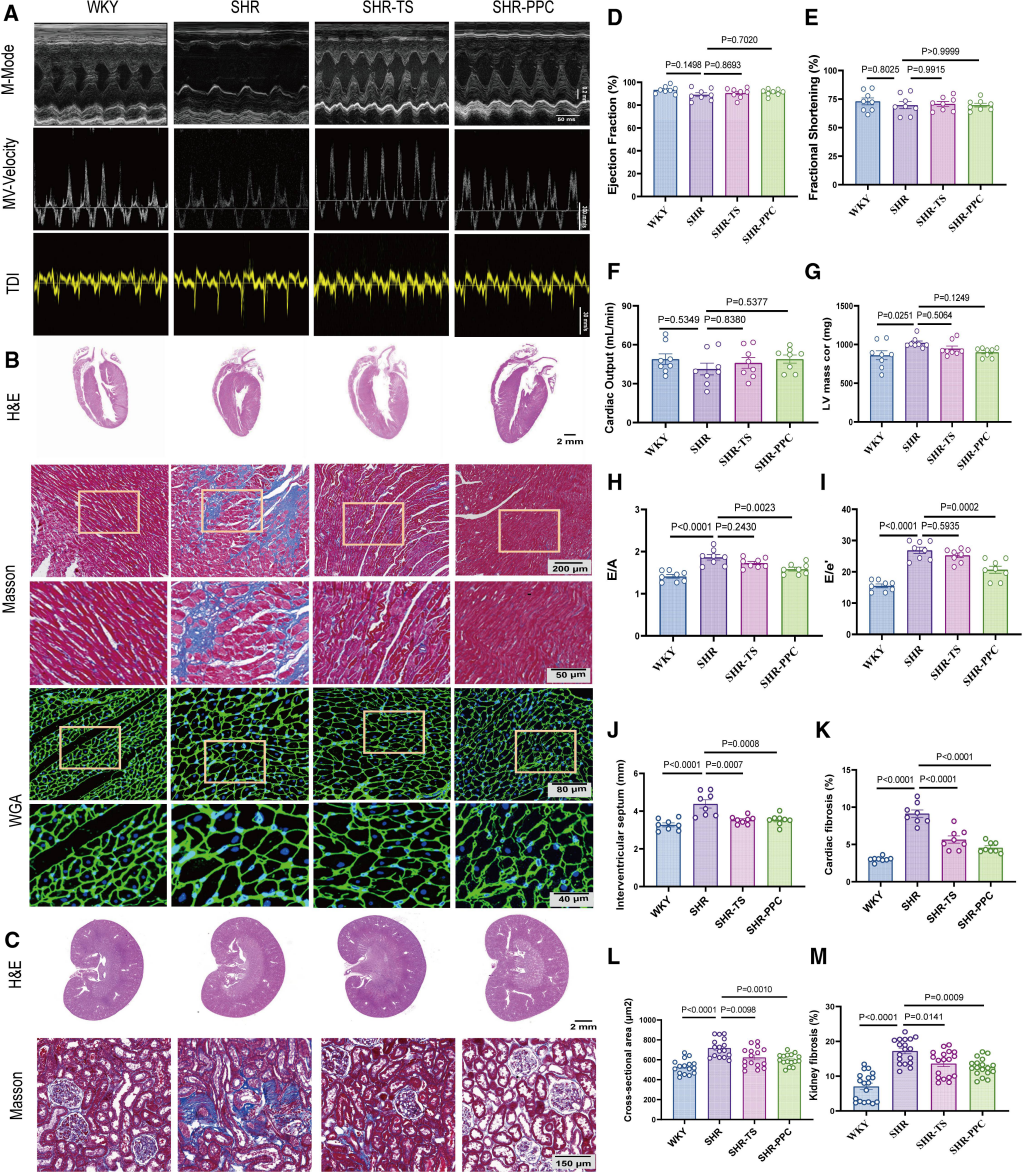
PPC attenuated cardiac and renal fibrosis and remodeling in SHR rats. **(A)** Representative images of M-mode (upper), pulse-wave Doppler (middle) and tissue Doppler (bottom) from WKY, SHR, SHR + TS or SHR + PPC groups. **(B)** Representative images of gross H&E staining (upper), Masson's trichrome staining (middle) and WGA staining (bottom); **(C)** representative photomicrographs of H&E staining with renal longitudinal section and Masson's trichrome staining with enlarged glomerular structures. Echocardiography examination of **(D)** ejection fraction, **(E)** fractional shortening, **(F)** cardiac output **(G)** left ventricular mass, **(H)** Mitral E/A ratio; **(I)** Mitral E/e′ ratio; **(J)** interventricular septum (IVS). **(K)** Quantitative analysis of heart fibrotic area (stained in blue). **(L)** WGA cross-sectional area, **(M)** quantitative analysis of kidney interstitial fibrosis.

We conducted echocardiographic assessments 12 weeks post-treatment. Compared to WKY rats, SHRs exhibited evident cardiac dysfunction, characterized by elevated left ventricular (LV) mass, E/A ratio, and E/e′ ratio (*p* < 0.05; Figures 2A,G–I), but without affecting ejection fraction, fractional shortening, and cardiac output (*p* > 0.05, Figures 2A,D–F). Following echocardiography, all rats were euthanized, and heart tissue cross-sections were stained with H&E or WGA. SHRs at 5 months old demonstrated increased cardiac fibrosis and hypertrophy in response to elevated blood pressure compared to WKY rats (*p* < 0.05, Figures 2B,J–L). However, 12 weeks of PPC treatment significantly attenuated both fibrosis and hypertrophy in SHRs, with effects comparable to TS treatment (Figures 2B,J–L). Additionally, PPC treatment mitigated kidney injury in SHRs, evidenced by reduced renal tubule-interstitial fibrosis (*p* < 0.05, Figure 2C,M).

### 3.3 PPC improved vascular dysfunction in SHRs

**Figure 3:**
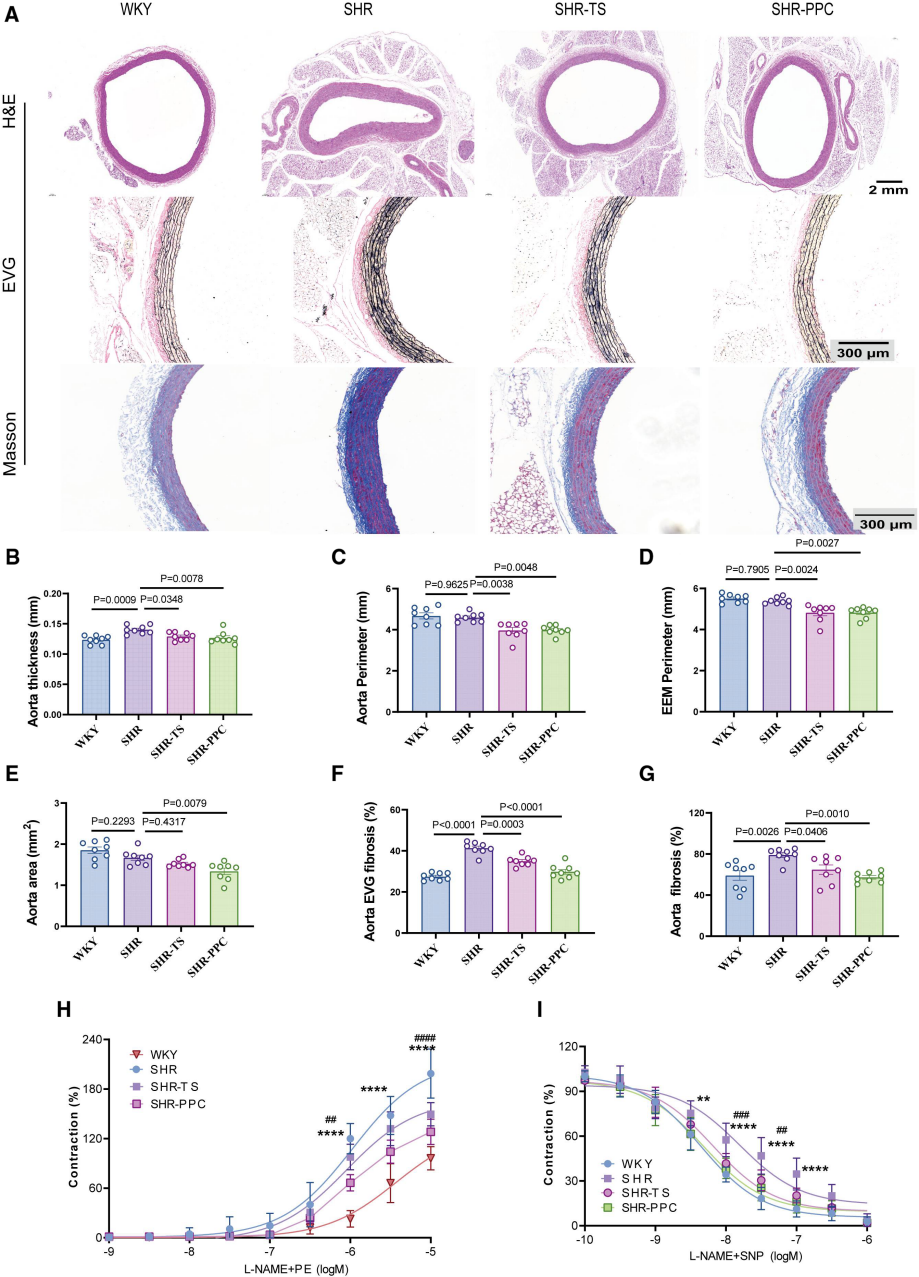
PPC inhibited vascular remodeling in SHR rats. **(A)** Representative H&E images of the cross section of aortas (upper), EVG staining (middle), and Masson's trichrome staining (bottom) of the enlarged aortic vessels. **(B)** Quantitative analysis of aorta thickness, **(C)** aorta perimeter, **(D)** External Elastic Membrane Perimeter (EEMP), **(E)** aortic area, **(F)** the elastic fiber area, and **(G)** aorta fibrosis. **(H,I)** The aortic rings were pretreated with PE(SNP) and vascular tension was determined. Data were presented as mean ± SD (n = 6–8 per group), One-way ANOVA with Bonferroni's *post hoc* test was performed to compare the difference among the multiple groups. PE, phenylephrine; SNP, sodium nitroprusside.

We investigated whether PPC treatment could ameliorate aortic function in SHRs. H&E staining revealed a decreased aortic area and an increased wall thickness (Figures 3A,B), while EVG staining demonstrated a disruption of the medial elastic lamina (Figures 3A,F), and Masson's trichrome staining indicated elevated aortic fibrosis in SHRs compared to WKY rats (Figures 3A,G). However, aortic area, perimeter, and external elastic membrane perimeter (EEMP) did not differ significantly between SHRs and WKY rats (Figures 3C–E). Following 12 weeks of PPC treatment, aortic fibrosis was significantly reversed. There were no significant differences in aortic area, wall thickness, elastin levels, or collagen production between PPC- and TS-treated SHRs (Figures 3B,F,G), suggesting that PPC's antihypertensive effects may mitigate the risk of aortic dysfunction.

We conducted aortic ring assays to further assess aortic function. Aortic rings isolated from SHRs and WKY rats were assessed for their ex vivo contractile response to phenylephrine (PE). Compared to WKY rats, SHRs exhibited significantly increased aortic contraction, which was partially inhibited by both PPC and TS treatments (Figure 3H). Similarly, aortic relaxation in response to sodium nitroprusside (SNP) was impaired in SHRs and partially restored by PPC or TS (Figure 3I).

### 3.4 PPC reduced inflammation, oxidative stress and disordered lipid metabolism in SHRs

**Figure 4:**
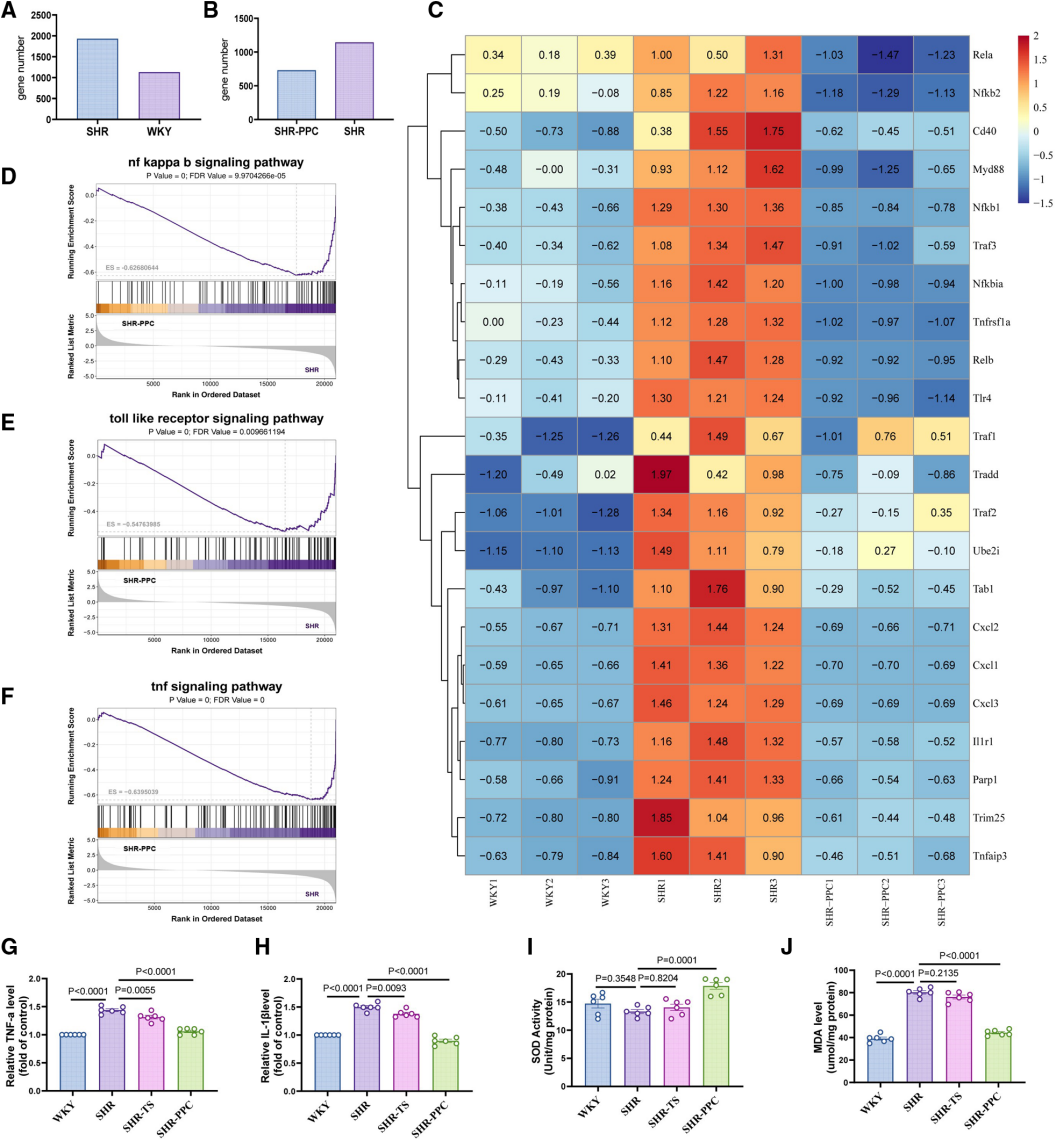
Bulk RNA-sequencing (RNA-seq) analysis was performed in the VSMCs from WKY rats, SHR rats treated with or without PPC. **(A,B)** The Bar chart of upregulated and downregulated genes numbers in SHR VSMCs vs. WKY VSMCs, or SHR-PPC VSMCs vs. SHR VSMCs. **(C)** Heatmap showed the significantly upregulated and downregulated genes among three groups of VSMCs. **(D–F)** Gene set enrichment analysis (GSEA) of the NF-κB, oxidative stress, and TNF-α signaling pathway. The *p* value and false discovery rate (FDR) are shown in each enrichment plot. **(G,H)** The circulating levels of TNF-α and IL-1β were determined by ELISA. **(I,J)** Oxidative stress in aortas was assessed by level of MDA and SOD. Data were presented as mean ± SD (*n* = 6 per group).

To explore the underlying mechanisms, we conducted RNA sequencing on aortic VSMCs from WKY rats, untreated SHRs, and SHRs treated with PPC. Compared to WKY-VSMCs, SHR-VSMCs exhibited 1,933 upregulated and 1,133 downregulated genes (Figure 4A), with PPC treatment markedly downregulating 1,144 genes in SHR aortic VSMCs (Figure 4B). Gene set enrichment analysis highlighted enrichment of these downregulated genes in inflammation, oxidative stress, and NF-*κ*B signaling pathways (Figures 4D–F). Heatmap analysis revealed significant upregulation of factors such as Rela, Relb, Myd88, Nfkb1, and Nfkb2 in SHR aortic VSMCs compared to WKY rats, most of which were attenuated following 12 weeks of PPC treatment (Figure 4C).

NF-*κ*B plays a crucial role in regulating inflammation and oxidative stress responses. To assess whether PPC's inhibition of NF-*κ*B contributed to reduced inflammation and oxidative stress in SHR aortas *in vivo*, we measured TNF-α and IL-1β levels in different rats. TNF-α and IL-1β were significantly elevated in SHRs aortas compared to WKY rats, however, PPC treatment effectively reduced these levels (Figures 4G,H). Additionally, MDA and SOD levels were evaluated in aortic tissues of WKY and SHR rats. Compared to WKY rats, SHR rats exhibited increased MDA and decreased SOD levels, both of which were significantly reversed following PPC treatment (Figures 4I,J). Interestingly, TS pretreatment did not show significant antioxidative effects in SHR-VSMCs, suggesting that PPC may effectively mitigate vascular oxidative injury.

### 3.5 Plpp3, the upstream target of the NF-κB pathway

**Figure 5:**
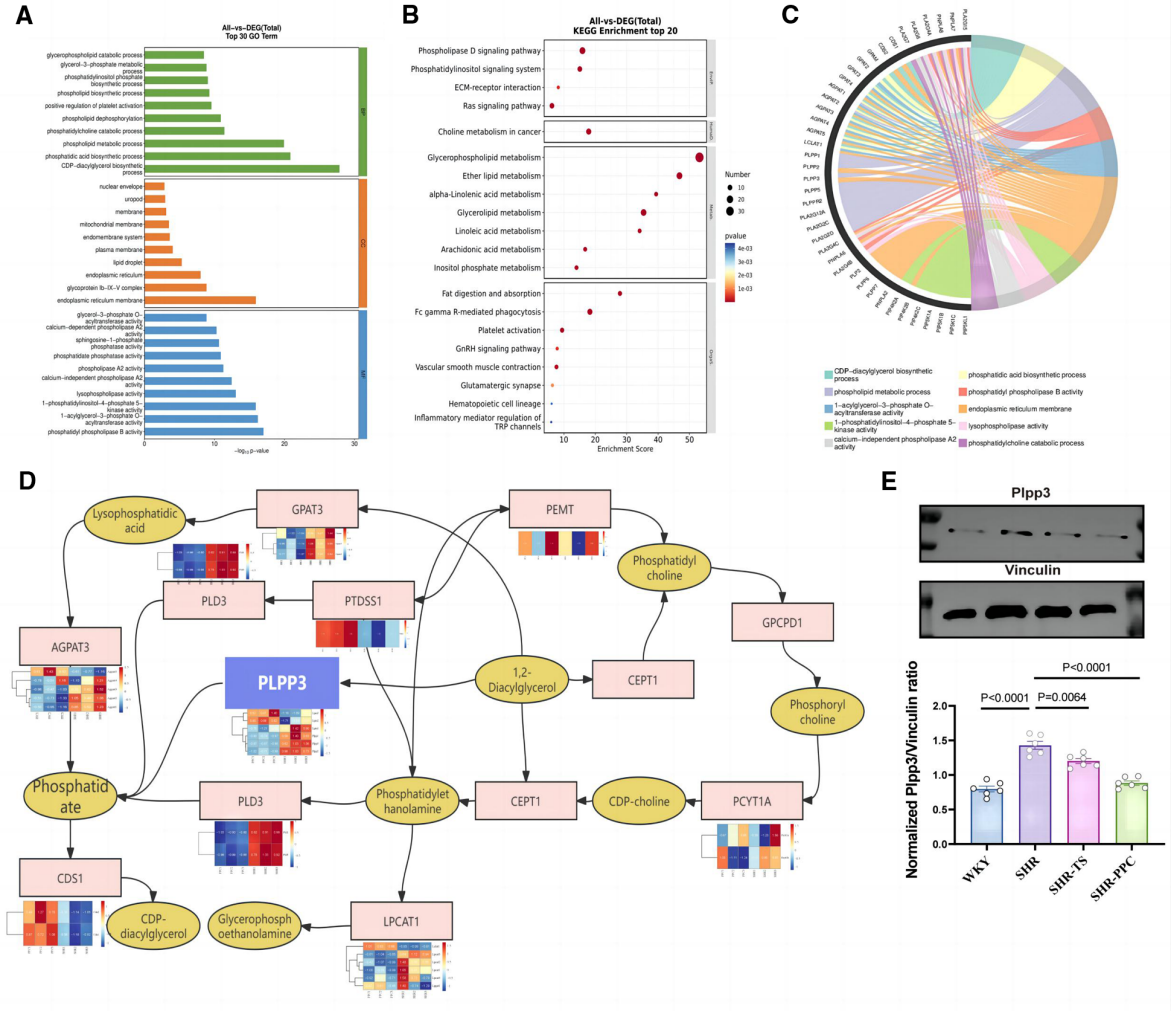
Plpp3 is the target for PPC-induced anti-inflammation and oxidative stress effect on SHR rats. **(A)** GO enrichment analysis of showed the top 30 DEGs in VSMCs between SHR-PPC and SHR groups. **(B)** The scatter plots of KEGG enrichment analysis showed that DEGs most significantly enriched in the top 20 signaling pathways. **(C)** Chord diagram showing the most enriched biological processes (GO terms) to explain the relationship between glycerophospholipid metabolism pathway and inflammation in SHR VSMCs following PPC treatment. **(D)** A center role of Plpp3 in the glycerophospholipid metabolism pathway during PPC treatment. **(E)** Plpp3 was inhibited by PPC in SHR VSMCs. Data were presented as mean ± SD (*n* = 6 per group).

To date, we have established that aortic VSMCs play a crucial role in modulating inflammatory and oxidative damage in SHRs, which can be influenced by PPC. However, the precise mechanism remains unclear. Using Gene Ontology (GO) and Kyoto Encyclopedia of Genes and Genomes (KEGG) analyses (Figures 5A,B), we observed that PPC treatment primarily affected pathways related to glycerophospholipid metabolism. Given PPC's profound impact on glycerophospholipid synthesis and metabolism, involving families such as phospholipase A2 (PLA2), Lipid phosphate phosphatases (LPPs), and phospholipid phosphatases (PLPPs), considering NF-*κ*B's role in regulating PLPPs expression via RelA and RelB transcription factors binding to their promoter regions, we conducted further analysis of PLA2s, PLPs, and PLPPs families in aortic VSMCs from SHR rats. We further identified significantly differentially expressed genes upstream and downstream of this pathway (Figures 5C,D), highlighting Plpp3 as a potential target suppressed by PPC. Consistently, PPC markedly inhibited the protein expression of Plpp3 in aortic VSMCs isolated from SHR rats (Figure 5E).

### 3.6 Overexpression of Plpp3 reversed aortic fibrosis, abolished PPC-mediated cardiorenal protection in SHRs

**Figure 6:**
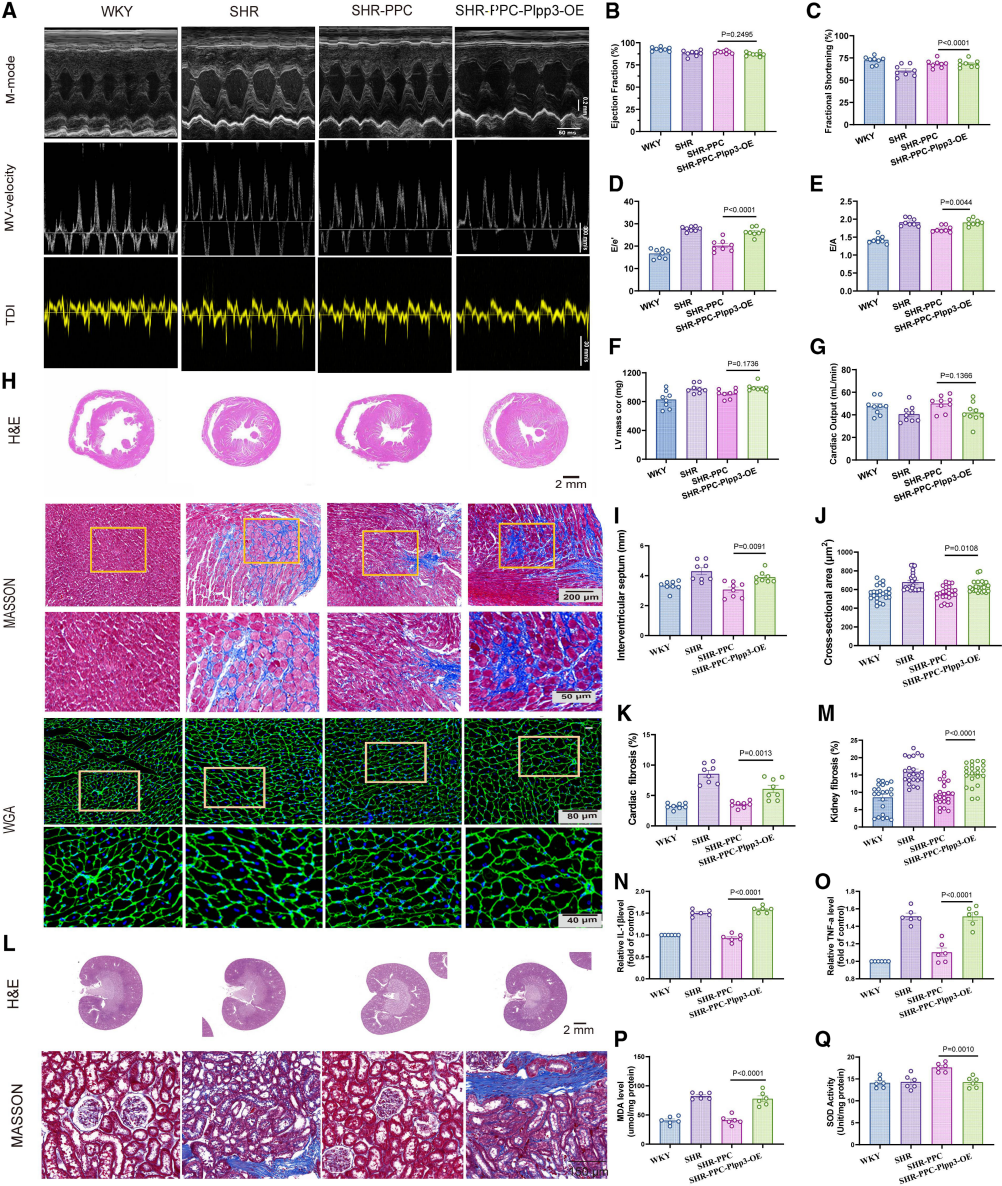
Overexpression of Plpp3 aggravated cardiorenal injury in PPC-treated SHR rats. **(A)** Representative M-mode (upper), pulse-wave Doppler (middle) and tissue Doppler (bottom) images, **(B)** Ejection fraction, **(C)** Fractional shortening, **(D)** Mitral E/e′ ratio, **(E)** Mitral E/A ratio, **(F)** LV mass, **(G)** Cardiac output were quantified. **(H)** Representative images of H&E, Masson's trichrome and WGA staining in cross sections of hearts from WKY rats, SHR rats, SHR-PPC rats or SHR-PPC rats with Plpp3 Overexpression. **(I–K)** Quantitative analysis of interventricular septum (IVS), cross sectional area, and cardiac fibrosis in different groups of rats, **(L)** Representative photomicrographs of H&E staining with renal longitudinal section and Masson's trichrome staining with enlarged glomerular structures. **(M)** Quantitative analysis of renal fibrosis. **(N,O)** The circulating levels of TNF-α and IL-1β were determined by ELISA. **(P,Q)** Oxidative stress in aortas was assessed by level of MDA and SOD. Data were presented as mean ± SD (*n* = 6 per group).

**Figure 7:**
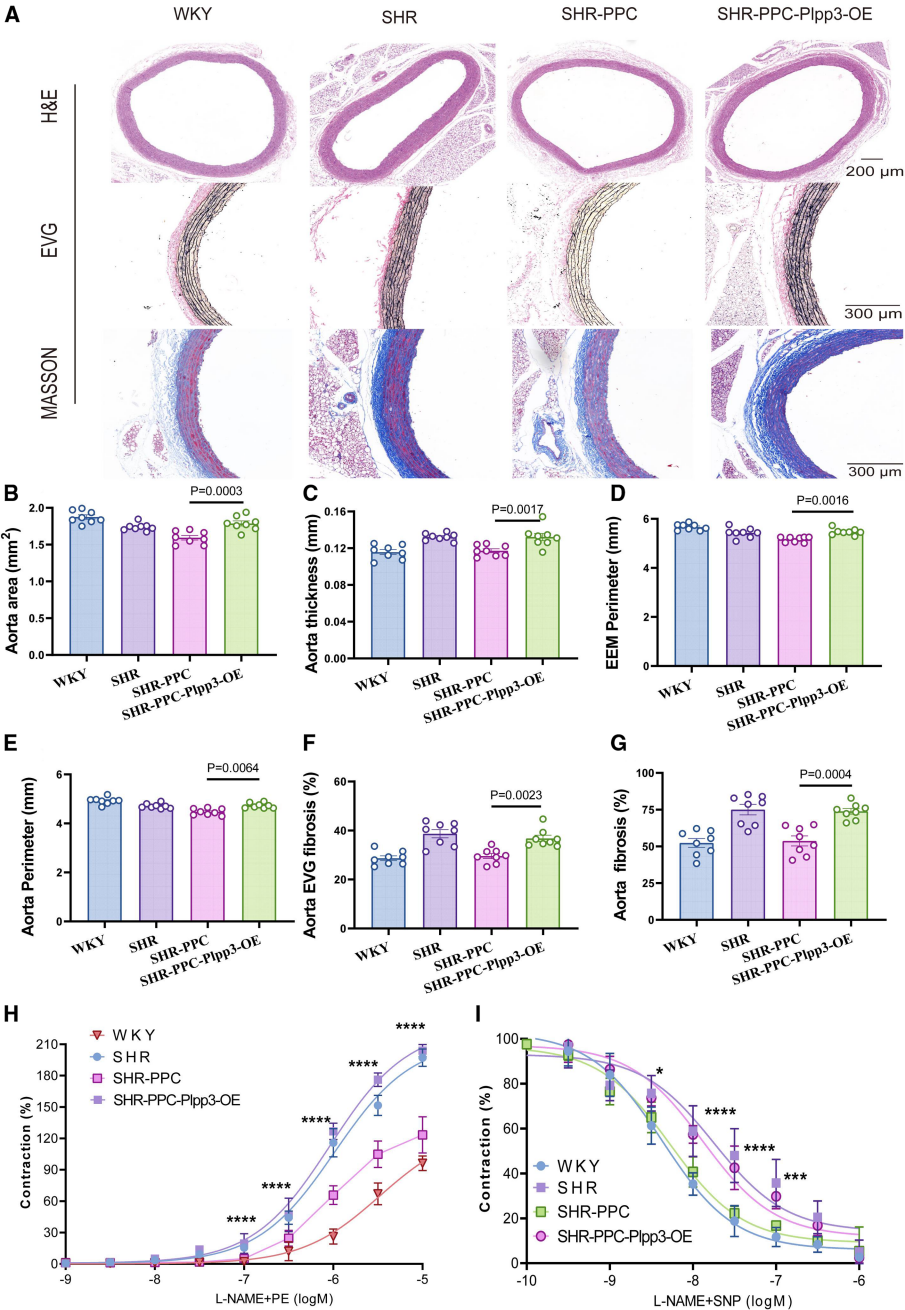
Overexpression of Plpp3 aggravated vascular remodeling in PPC treated SHR rats. **(A)** Representative images of H&E, EVG and Masson's trichrome staining. **(B)** Quantitative analysis of aortic area, **(C)** aorta thickness, **(D)** External Elastic Membrane Perimeter (EEMP), **(E)** aorta perimeter, **(F)** Elastic fiber area in the thoracic aorta, **(G)** aortic fibrosis. **(H,I)** The aortic rings were pretreated with PE (phenylephrine) sodium nitroprusside (SNP), and vascular tension was determined. Data were presented as mean ± SD (*n* = 6–8 per group), One-way ANOVA with Bonferroni's post hoc test was performed to compare the difference among the multiple groups.

Given the pivotal role of the Plpp3-mediated NF-*κ*B pathway in vascular inflammation and remodeling in SHR rats, we examined whether Plpp3 overexpression could counteract PPC's protective effects on aortic injury in SHRs. Plpp3 overexpression in SHR rats using AAV9 was confirmed via Western blot (data not shown). This overexpression notably exacerbated cardiac dysfunction in PPC-treated SHR rats, evidenced by decreased EF and FS values (Figures 6A–C), increased LV mass (Figures 6A,F), altered E/A and E/e′ ratios (*p* < 0.05; Figures 6A,D,E), and reduced cardiac output (*p* < 0.05; Figure 6G). Following Plpp3 overexpression, both cardiac hypertrophy and fibrosis were heightened in PPC-treated SHR rats (Figures 6H–J). Moreover, renal fibrosis and injury were significantly exacerbated by Plpp3 overexpression (Figures 6K–M). As expected, inflammation (Figures 6N,O) and oxidative stress (Figures 6P,Q) were increased in the aortas of PPC-treated SHR rats following Plpp3 overexpression. Therefore, overexpressing Plpp3 worsened aortic fibrosis and dysfunction in PPC-treated SHRs, along with notable impairment of vascular tone (Figure 7). Plpp3 overexpression significantly increased wall thickness (Figures 7A,B), degradation of the medial elastic lamina in the aortas (Figures 7A,C), and aortic fibrosis (Figures 7A,D) in PPC-treated SHR rats. In addition, improvement of vascular remodeling by PPC treatment was also abolished by Plpp3 overexpression, indicating by increased aortic area (Figure 7E), aortic perimeter (Figure 7F) and EEMP (Figure 7G). Vascular tension was also significantly impaired after Plpp3 overexpression (Figures 7H,I). These findings suggest that Plpp3 is a upstream target of NF-*κ*B signaling in hypertensive vascular injury, and PPC may confer protection by modulating Plpp3 activity.

## 4 Discussion

PPC was initially used in treating liver diseases (11). Recently, PPC's role has expanded to include organ protection, such as alleviating synovial inflammation via reduced ROS production and preservation of mitochondrial membrane potential (13). PPC reduced arthritis inflammation by inhibiting LPS-induced pro-inflammatory macrophage polarization (14). Moreover, PPC suppressed gastric cancer by activating HMOX1-mediated ferroptosis (15). Significantly, PPC's primary function is its antioxidative stress effect. In this study, we investigated the role of PPC in regulating hypertension and hypertensive-related organ damage. Consistently, we observed that PPC effectively reduced ROS production in the aortas of SHRs, as evidenced by decreased MDA levels and increased SOD expression. Furthermore, after 12 weeks of PPC treatment, blood pressure significantly decreased to 180/130 mmHg in SHR rats compared to 240/160 mmHg in those treated with saline alone, suggesting that PPC (200 mg/kg/day) was effective in lowering blood pressure.

We found that there is a lack of significant changes in cardiac function among SHRs with or without treatment with TS or PP. The reasons can be understood from the following considerations. Firstly, there is a certain time window between the cardiac ultrasound examination and the actual sampling time. Additionally, morphological changes in hearts typically occur earlier than changes in cardiac function. For example, there is no notable discrepancy in EF values between those HFpEF patients and the general population. However, cardiac hypertrophy and fibrosis are frequently observed in individuals with HFpEF. Our study yielded similar results, with no change in cardiac LVEF observed between SHR and WKY rats, or among SHRs with or without treatment of TS or PPC. However, the ratios of E/A and e/e′ were both significantly higher in SHRs than in WKY rats, while these two indices were significantly lower in SHRs with PPC treatment. These findings suggest that these two indices may be sensitive parameters for the examination of SHRs.Secondly, it is recognized that SHRs are known to exhibit compensatory mechanisms in response to long-term hypertension, which has the potential to mask true pathological cardiac states. It has been reported that compensatory hypertrophy is a common occurrence in SHRs (16, 17), and that it can persist for a significant period of time before progressing to decompensatory hypertrophy and heart failure during the development of hypertension. Furthermore, resveratrol has been shown to prevent the development of pathological hypertrophy and contractile dysfunction in SHRs without affecting blood pressure (18). This literature may explain the lack of significant changes in cardiac function among SHRs with or without treatment with TS or PPC due to the relatively weak effect of blood pressure.Thirdly, the relatively small number of rat samples used in the study may not be sufficient to generate statistically significant differences. Increasing the number of rats in future experiments may help clarify these differences.

The increase in MDA and the decrease in SOD usually indicate oxidative stress (19). Oxidative stress is a key mechanism in hypertensive vascular diseases (20). For instance, cocaine exposure in mice increases blood pressure and aortic stiffness, correlating with elevated ROS levels in the aortas, a phenomenon similarly observed in hypertensive patients (21). Furthermore, reducing ROS production by inhibiting endothelial Nox2 protects mice from AngII-induced vascular oxidative stress (22, 23). An increase in ROS generation is associated with cell death and organ injury. We found PPC effectively reduced aortic MDA production but increased SOD generation in SHR rats after a treatment lasting for 12 weeks. We also found that cardiac and kidney fibrosis were both inhibited in SHR rats by PPC treatment. Evidence suggests that ROS-induced NF-*κ*B signaling plays a pivotal role in hypertensive vascular remodeling by enhancing cell adhesion, proliferation, and migration (24). Through RNA sequencing, we identified significant upregulation of several genes involved in the NF-*κ*B pathway—Rela, Myd88, and Nfkb1—in VSMCs of SHRs compared to WKY rats. Treatment with PPC significantly downregulated all these genes. This suggests that PPC effectively inhibits proliferation and migration of aortic VSMCs by antagonizing ROS-dependent NF-*κ*B signaling, potentially preventing aortic fibrosis and remodeling.

Why might a crucial component in membrane phospholipid synthesis confer protective effects against arterial remodeling induced by heightened ROS-dependent NF-*κ*B activation? We analyzed RNA sequencing data. We found significant differences in processes related to glycerophospholipid metabolism between PPC-treated and untreated aortic VSMCs in SHRs. Notably, the PLA2 and Plpp3 gene families stood out among the differentially expressed genes in SHR-VSMCs following PPC treatment. Previous studies demonstrated that LPA enhances the binding of NF-*κ*B family transcription factors, such as RelA and RelB, to the Plpp3 promoter, thereby increasing Plpp3 transcription (25, 26). Therefore, we hypothesized that PPC attenuated vascular oxidative stress and inflammation by targeting Plpp3 (27, 28). Overexpressing Plpp3 in VSMCs increased ROS production and secretion of TNF-α and IL-1β, counteracting PPC's protective effects on SHR aortas.

Limitations of the study warrant attention. Despite its widespread use in hypertension research, the rat model presents inherent discrepancies in anatomy, physiology, and pathophysiology compared to humans, potentially limiting direct applicability to human conditions. Secondly, our study exclusively examined male subjects, potentially introducing bias by disregarding gender differences in the experimental outcomes. Thirdly, we also only found that PPC affects VSMCs and did not study their effects on other cells. Further research is required to confirm whether the effective 200 mg/kg/day dosage used in our *in vivo* SHRs experiments applies to human hypertension-related vascular injury or remodeling, and to elucidate the underlying mechanisms.

**Figure 8:**
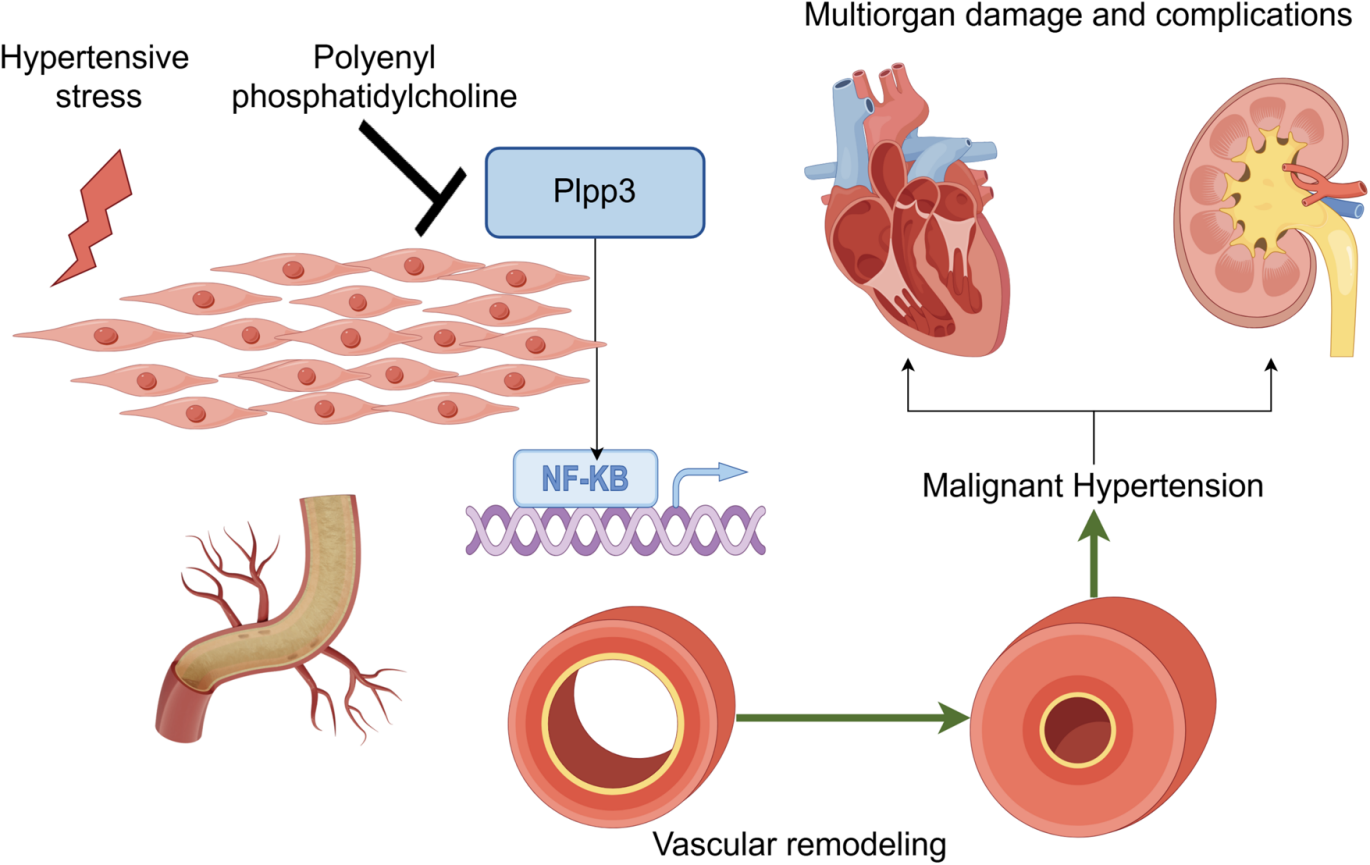
PPC mitigates aortic fibrosis, remodeling and cardiorenal injury by modulating Plpp3/Nf-kB pathway (by figdraw).

In conclusion, our study offers new evidence supporting PPC's protective effect against hypertensive disease (Figure 8). PPC treatment lowers blood pressure in SHRs, alleviates cardiac and renal fibrosis, and diminishes oxidative stress and inflammatory responses. RNA sequencing identifies Plpp3 as a key target of PPC, indicating its potential as a therapeutic target for hypertension-related diseases.

## Data Availability

The original contributions presented in the study are publicly available. The relative data were submitted to NCBI under BioProject accession no. PRJNA1148940, this data can be found here: https://www.ncbi.nlm.nih.gov/bioproject/PRJNA1148940.
