# Supplementary material for: Polyenylphosphatidylcholine alleviates cardiorenal fibrosis, injury and dysfunction in spontaneously hypertensive rats by regulating Plpp3 signaling
Source: Front Cardiovasc Med. 2024 Sep 30;11:1458173. doi: 10.3389/fcvm.2024.1458173 (PMC11472324; doi:10.3389/fcvm.2024.1458173)
Supplement: Supplementary file 1 [file Datasheet1.docx]

**Methods**

**Chemicals and Reagents and antibodies**

PPC was obtained from Beijing Sanofi Pharmaceutical Co., Ltd. (Beijing, China), and TS (80 mg/tablet) was supplied by Boehringer Ingelheim Pharma GmbH &amp; Co. KG. Antibodies used included anti-Plpp3 (A24734, 1:500 dilution) from ABclonal Technology (China), anti-SM22α (ab10135, 1:200 dilution) from Abcam (China), anti-α-SMA (#19245, 1:1000 dilution), and anti-Vinculin (#13901, 1:1000 dilution) from Cell Signaling Technology (USA).

**Experimental Animals**

Eight-week-old Wistar Kyoto (WKY) rats and SHRs were sourced from Beijing Vital River Laboratory Animal Technology Co. Ltd (Beijing, China) and housed in a controlled environment with ad libitum access to tap water. Following a one-week acclimation period, rats were randomly allocated into groups (n=8 per group): WKY rats receiving normal saline; SHRs receiving normal saline or varying doses of PPC (150mg/kg/day, 200mg/kg/day, 250mg/kg/day) or TS(10mg/kg/day). Additionally, an AAV-Plpp3-OE transfection group was included for rescue experiments (n=8). The study spanned 12 weeks, with biweekly blood pressure measurements and echocardiography performed post-treatment. Following euthanasia under anesthesia (intraperitoneal injection of 0.3% pentobarbital sodium (50 mg/kg)), cardiac, kidney, and aortic tissues were collected for further analysis. Experimental protocols were approved by the Institutional Animal Use and Care Committees of Zhongshan Hospital (Shanghai, China) and adhered to NIH Guidelines.

**Blood pressure measurement**

We utilized a CODA tail-cuff device (Kent Scientific, Torrington, CT, USA) for non-invasive monitoring of conscious rats' systolic/diastolic blood pressure (SBP/DBP) and mean blood pressure (MBP) [15]. Measurements were conducted under stable conditions, with a minimum of ten readings per session. Rats underwent a 3-5 day acclimation period prior to measurements to reduce potential external influences on blood pressure values.

**Echocardiographic assessment**

Cardiac structure and function were evaluated using 2-D guided M-mode echocardiography (Vevo 2100, Visualsonics, Toronto, ON, Canada). Fractional shortening (FS) was calculated as (LVEDD - LVESD)/LVEDD from left ventricular (LV) end-diastolic diameter (LVEDD) and LV end-systolic diameter (LVESD). Ejection fraction (EF), heart rate (HR), and additional parameters such as peak Doppler blood inflow velocities across the mitral valve in early and late diastole, peak tissue Doppler myocardial relaxation velocity at the mitral valve annulus in early diastole, and early filling deceleration time were also recorded[16].

**Examination of mesenteric artery tension**

The contractile properties of the second-order branches of the mesenteric artery were evaluated using a Multi Myograph System (Model 610 M, Danish Myo Technology A/S, Aarhus N, Denmark). Following animal sacrifice, arteries were rapidly dissected and placed in ice-cold Krebs solution. After removal of adherent connective tissue, artery segments approximately 2-3 mm in length were prepared and suspended in the organ chamber of the Multi Myograph System, oxygenated with 95% O2 and 5% CO2. After achieving stable resting tension, arteries were stimulated with 80 mmol/L KCl to confirm contractile function. Subsequently, arteries were exposed to L-nitro-argininemethyl ester (L-NAME, 100 µmol/L) for 30 minutes to assess endothelium-independent relaxations or contractions. Contractile responses were induced by phenylephrine (PE), while sodium nitroprusside (SNP) was used to induce endothelium-independent relaxations.

**Histological examination**

Following pentobarbital sodium anesthesia, rats were euthanized, and samples of the thoracic aorta, heart, and kidney were promptly fixed in 4% paraformaldehyde (Beyotime Biotech Inc, China). Tissues were sequentially dehydrated, cleared, and embedded to produce 5-µm thick paraffin sections. Hematoxylin and eosin (H&E) staining was performed accordingly. Vessel thickness was measured at three uniformly selected positions within each field of view, and the average of these measurements was used to determine vessel wall thickness. Aortic diameter was calculated based on the perimeter, and differences in vessel wall thickness and wall-to-diameter ratio among groups were compared. Masson's trichrome staining was utilized to visualize collagen fibers, which appear blue under microscopy, for subsequent quantitative analysis. Elastic fibers were stained using Verhoeff's and Van Gieson's solutions in EVG staining, where they appear dark or black. Frozen cardiac cross sections were stained with wheat germ agglutinin (WGA) to measure cardiomyocyte cross-sectional area [17]. Imaging and quantitative analysis were performed using a digital microscope (×400) and ImageJ Fiji software (version 2.3.0, NIH).

**Bulk RNA-Seq and Bioinformatics Analysis**

Total RNA from VSMCs was extracted using a commercial kit (Cat No. 74104, QIAGEN) following the manufacturer's protocol. RNA-seq and bioinformatic analysis were performed at Biotree Biotech Co.Ltd. (Shanghai, China).

**VSMCs cell culture**

VSMCs were obtained from the thoracic aortas of 5- to 6-week-old SHR/WKY rats, following established protocols with minor adaptations[18]. Aortas were carefully dissected post-rat euthanasia, with precise incisions made to isolate the thoracic segments prior to washing with ice-cold PBS. Under microscopic guidance, the adventitia was gently removed, and the aortas were longitudinally opened to scrape off endothelial cells. Small aortic sections were cultured in dishes, and migrating cells were harvested for subsequent passages. VSMCs from the third passage were used, confirmed for purity via staining with anti-SM22α and anti-α-SMA antibodies. Dulbecco’s Modified Eagle Medium (DMEM) supplemented with 10% fetal bovine serum and 1% penicillin-streptomycin was used, and cells at 70% confluence were treated with or without PPC (25 uM) for 48 hours in experiments.

**adeno-associated virus (AAV9) delivered Plpp3-OE virus injection**

The AAV9 virus, developed by Hanbio Biotechnology (Shanghai, China), was utilized in this study. The AAV9 vectors carrying the Plpp3 Coding sequence (CDS, Chromosome 5, NC_086023.1) with an α-SM22 promoter (pAAV9-α-SM22-Plpp3-OE) or a negative control virus (pAAV9-α-SM22-NC) were administered to SHR rats via tail vein injection at a dose of 1 × 10^12^ μg/ml (100 μl per rat). After 12 weeks, aortic tissues from AAV9 transfected rats were collected to evaluate the overexpression of Plpp3 *in vivo*.

**Western blotting analysis**

Tissue or cultured cell proteins were extracted using lysis buffer according to the manufacturer's instructions. Total protein concentrations were determined using a BCA Protein Assay Kit (Thermo Scientific). Equal protein amounts from samples were loaded onto SDS-PAGE gels and transferred to PVDF membranes. Following blocking with 5% nonfat dry milk in Tris buffered Saline-Tween (TBST) for 1 hour at room temperature, membranes were incubated overnight at 4℃ with specific primary antibodies. After three TBST washes, membranes were exposed to HRP-conjugated secondary antibodies for 1 hour at room temperature. Protein bands were visualized using ECL western blotting substrate (Cat No. WBLUF0500, MilliporeSigma). Images were analyzed using Image Lab 3.0 and ImageJ software. Protein levels were normalized to Vinculin and expressed as a percentage relative to untreated controls.

**Measurement of oxidative stress**

The aortic tissue was homogenized using a tissue-lyser apparatus and stored at -80℃. Levels of malondialdehyde (MDA) (S0131S, Beyotime, Shanghai, China) and superoxide dismutase (SOD) activity (S0109, Beyotime, Shanghai, China) were subsequently measured using colorimetric assays with standard kits.

**Enzyme-linked immunosorbent assay (ELISA)**

Upon sacrifice, serum samples were collected from rats. Levels of circulating TNF-α (PT516, Beyotime, Shanghai, China) and IL-1β (PI303, Beyotime, Shanghai, China) were quantified using ELISA Kits following the manufacturers' protocols.

**Statistical Analysis**

Continuous variables were presented as mean ± standard deviation. Normality of data distribution was assessed using the Shapiro–Wilk test. For normally distributed data, comparisons between two groups were performed using independent sample Student's t-tests, while comparisons among multiple groups were conducted using one-way analysis of variance (ANOVA), followed by Tukey's post-hoc tests. Graphs were created using GraphPad Prism 9.3.0. Bonferroni correction was applied to adjust for multiple comparisons and maintain a stringent overall error rate. Statistical significance was set at P＜ 0.05, and all tests were two-tailed.
