# Supplementary figures and images for: Polyenylphosphatidylcholine alleviates cardiorenal fibrosis, injury and dysfunction in spontaneously hypertensive rats by regulating Plpp3 signaling
Source: Front Cardiovasc Med. 2024 Sep 30;11:1458173. doi: 10.3389/fcvm.2024.1458173 (PMC11472324; doi:10.3389/fcvm.2024.1458173)

plpp3 典型图

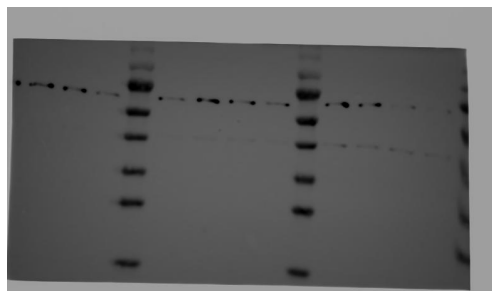

plpp3 统计图1

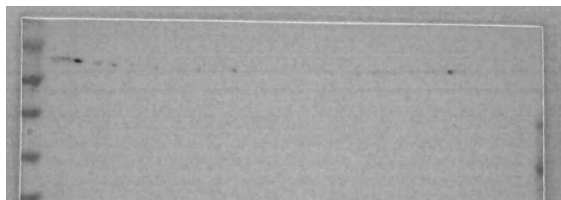

Vinculin 统计图1

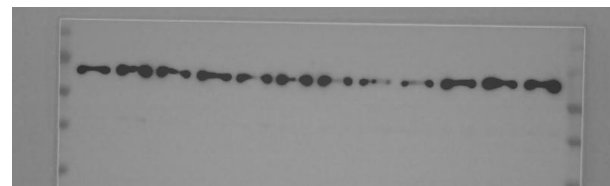

内参 Vinculin 典型图

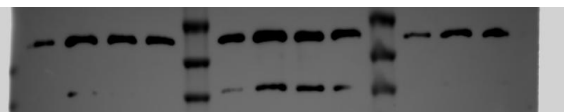

plpp3 统计图2

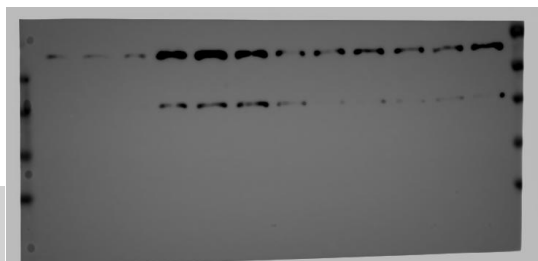

Vinculin 统计图2

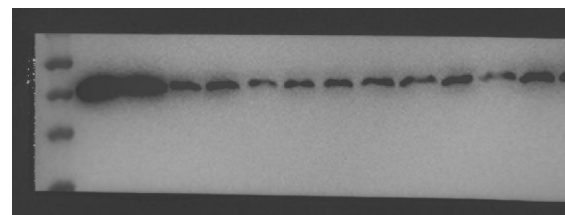

plpp3 统计图3

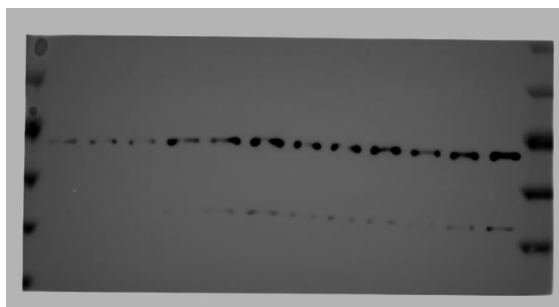

Vinculin 统计图3

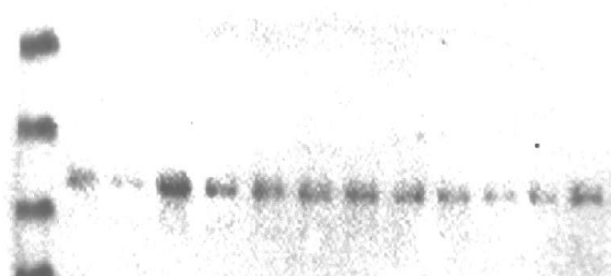

Supplement: Supplementary file 3 [file Image1.pdf]
